# Salidroside inhibits platelet function and thrombus formation through AKT/GSK3β signaling pathway

**DOI:** 10.18632/aging.103131

**Published:** 2020-04-30

**Authors:** Guangyu Wei, Xiaoqi Xu, Huan Tong, Xiamin Wang, Yuting Chen, Yangyang Ding, Sixuan Zhang, Wen Ju, Chunling Fu, Zhenyu Li, Lingyu Zeng, Kailin Xu, Jianlin Qiao

**Affiliations:** 1 Blood Diseases Institute, Xuzhou Medical University, Xuzhou, China; 2 Department of Hematology, The Affiliated Hospital of Xuzhou Medical University, Xuzhou, China; 3 Key Laboratory of Bone Marrow Stem Cell, Xuzhou, China

**Keywords:** salidroside, platelet, thrombus formation, AKT, GSK3β

## Abstract

Salidroside is the main bioactive component in *Rhodiola rosea* and possesses multiple biological and pharmacological properties. However, whether salidroside affects platelet function remains unclear. Our study aims to investigate salidroside’s effect on platelet function. Human or mouse platelets were treated with salidroside (0-20 μM) for 1 hour at 37°C. Platelet aggregation, granule secretion, and receptors expression were measured together with detection of platelet spreading and clot retraction. In addition, salidroside (20 mg/kg) was intraperitoneally injected into mice followed by measuring tail bleeding time, arterial and venous thrombosis. Salidroside inhibited thrombin- or CRP-induced platelet aggregation and ATP release and did not affect the expression of P-selectin, glycoprotein (GP) Ibα, GPVI and α_IIb_β_3_. Salidroside-treated platelets presented decreased spreading on fibrinogen or collagen and reduced clot retraction with decreased phosphorylation of c-Src, Syk and PLCγ2. Additionally, salidroside significantly impaired hemostasis, arterial and venous thrombus formation in mice. Moreover, in thrombin-stimulated platelets, salidroside inhibited phosphorylation of AKT (T308/S473) and GSK3β (Ser9). Further, addition of GSK3β inhibitor reversed the inhibitory effect of salidroside on platelet aggregation and clot retraction. In conclusion, salidroside inhibits platelet function and thrombosis via AKT/GSK3β signaling, suggesting that salidroside may be a novel therapeutic drug for treating thrombotic or cardiovascular diseases.

## INTRODUCTION

Platelets are well known regulators in the pathological thrombosis and physiological hemostasis. When there is an injury of the vessel wall, collagen and von Willebrand factor (VWF) will be exposed from the sub-endothelial matrix. Circulating platelets will adhere and attach to the damaged area through binding to collagen and VWF by glycoprotein (GP)VI and GPIbα [1–3]. Occupancy of these platelet receptors stimulates the transduction of intraplatelet signaling pathway, resulting in integrin α_IIb_β_3_ activation (inside-out signaling), which regulates platelet aggregation via binding to fibrinogen, fibronectin or VWF [4, 5]. At the same time, ligands binding to α_IIb_β_3_ also trigger several intracellular signaling events (outside-in signaling), leading to tyrosine phosphorylation of multiple signaling proteins, such as c-Src, spleen tyrosine kinase (Syk), phospholipase Cγ2 (PLCγ2), which participate in mediating platelet spreading, clot retraction and stabilization of thrombosis [6].

As a small genus of the Crassulaceae family, *Rhodiola rosea* L. has been widely used as a botanical medicine for a long time for prevention and treatment of multiple diseases, such as fatigue, pains, Alzheimer’s disease, depression, and anxiety [7, 8]. In addition, it is also used as a cardiopulmonary protective agent in traditional folk medicine [9]. Several recent studies have demonstrated the potential applications of *Rhodiola* extracts in preventing cardiovascular diseases and cancer [10–12]. Till now, several specialized glycosides have been identified, including rosiridin, rhodionin, rosarin, rosin, rosavin, and salidroside [11]. Salidroside is the main bioactive component in *Rhodiola rosea* and possesses several biological and pharmacological properties, such as anti-inflammatory, anti-oxidative, anti-aging, anti-cancer, anti-depressant, neuroprotective, and hepatoprotective activities [13, 14]. In addition, salidroside has been shown to reduce blood pressure and alleviate cerebrovascular contractile activity in diabetic Rats [15], and attenuate oxidized low-density lipoprotein-induced endothelial cell injury [16] or vascular endothelial dysfunction [17]. Furthermore, salidroside has also been demonstrated to decrease atherosclerotic plaque formation in mice with deficiency of low-density lipoprotein receptor [18] and ameliorate chronic hypoxia-induced pulmonary arterial hypertension in mice [19]. However, whether salidroside plays a role in platelet function is unclear.

In the present study, through treating platelets with salidroside, we aim to investigate the effect of salidroside on platelet aggregation, activation, spreading and clot retraction. Moreover, salidroside’s effect on *in vivo* hemostasis and thrombosis was also evaluated.

## RESULTS

### Salidroside inhibits human platelet aggregation and ATP release

**Figure 1:**
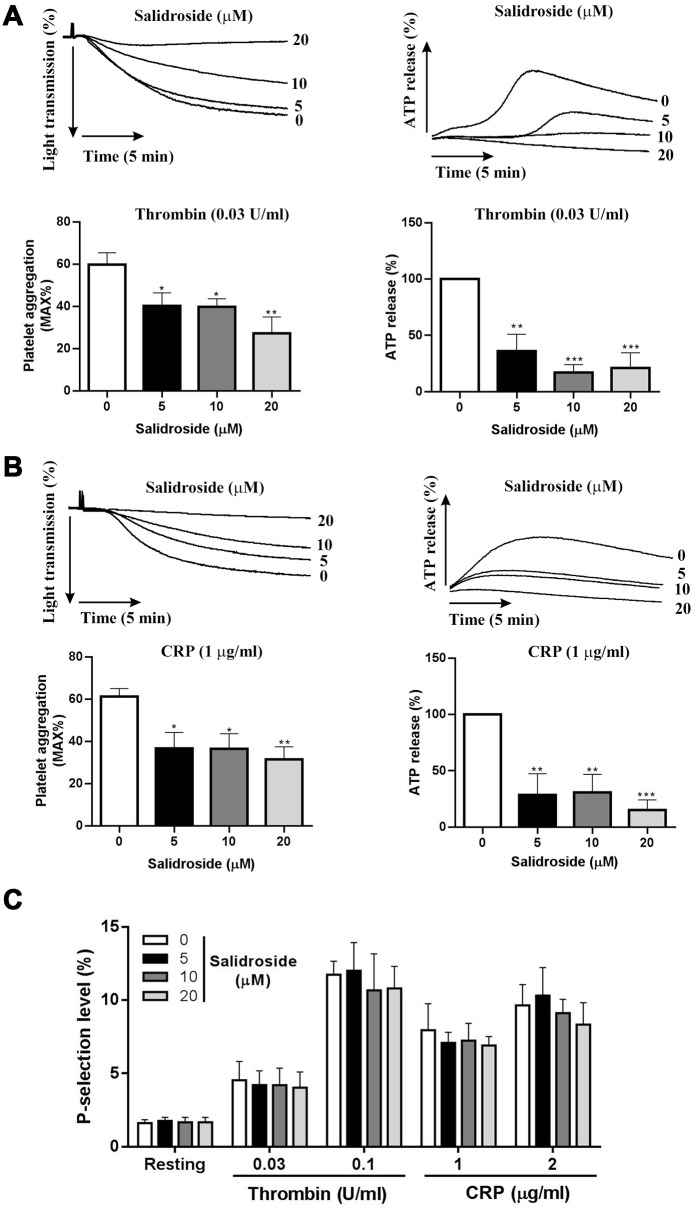
**Platelet aggregation and ATP release.** Washed human platelets were treated with salidroside (0, 5, 10 and 20 μM) at 37°C for 1 h and platelet aggregation and ATP release was measured after stimulation with thrombin (0.03 U/ml) (**A**) or CRP (1 μg/ml) (**B**) in a Lumi-Aggregometer. Meanwhile, P-selectin expression was measured by flow cytometry (**C**). Data were presented as mean ± SE (n=4-6) and analyzed by one-way ANOVA. Compared to 0, *P < 0.05; **P < 0.01; ***P < 0.001.

Through incubation with human washed platelets with salidroside (0, 5, 10 and 20 μM), we investigated whether salidroside affects platelet aggregation in response to thrombin (0.03 U/ml) or CRP (1 μg/ml) stimulation. As seen in Figure 1, salidroside treatment significantly reduced thrombin (Figure 1A) or CRP (Figure 1B)-induced platelet aggregation compared with vehicle treatment (0 μM salidroside) with more decrease of platelet aggregation after treatment with the highest concentration of salidroside (20 μM). To further investigate whether salidroside influences ATP release which simultaneously occurs along with platelet aggregation, we also detected ATP release and found significantly reduced ATP release from thrombin or CRP-stimulated platelets after salidroside treatment compared with vehicle treatment (Figure 1A, 1B), with more reduction being observed in platelets treated with the highest dose of salidroside (20 μM). As alpha-granule content is also released after platelet aggregation, we further measured platelet alpha-granule content release (surface P-selectin expression) after salidroside treatment. Surprisingly, salidroside did not affect thrombin or CRP-induced platelet alpha-granule content release even at a highest concentration (20 μM) as shown by no changes of platelet P-selectin surface expression after salidroside treatment compared with vehicle (Figure 1C). This difference might be due to the different function of alpha granules and dense granules [20, 21], and ATP or ADP secretion from dense granules has been reported to promote platelet in response to low level of agonists [22].

### No change of expression of human platelet glycoprotein receptors after salidroside treatment

**Figure 2:**
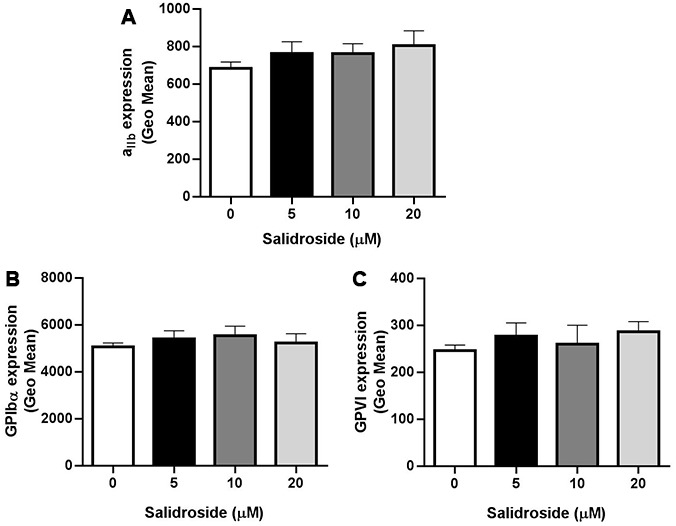
**Expression of platelet glycoprotein receptors.** After treatment with salidroside, the expression of α_IIb_β_3_ (**A**), GPIbα (**B**) and GPVI (**C**) was detected by flow cytometry. Data were presented as mean ± SE (n=3-4) and analyzed by one-way ANOVA.

Platelet glycoprotein receptors GPIbα, GPVI and GPIIb/IIIa (α_IIb_β_3_) play critical roles in regulating platelet aggregation and function [23, 24]. Since platelet aggregation was inhibited after salidroside treatment, we next evaluated whether salidroside affects platelet glycoprotein receptors and found that salidroside did not influence the surface expression of α_IIb_β_3_ (Figure 2A), GPIbα (Figure 2B) and GPVI (Figure 2C) even at a higher concentration as no significant changes of the expression of these platelet receptors were observed after salidroside treatment compared with vehicle.

### Salidroside inhibits human platelet α_IIb_β_3_ outside-in signaling transduction

**Figure 3:**
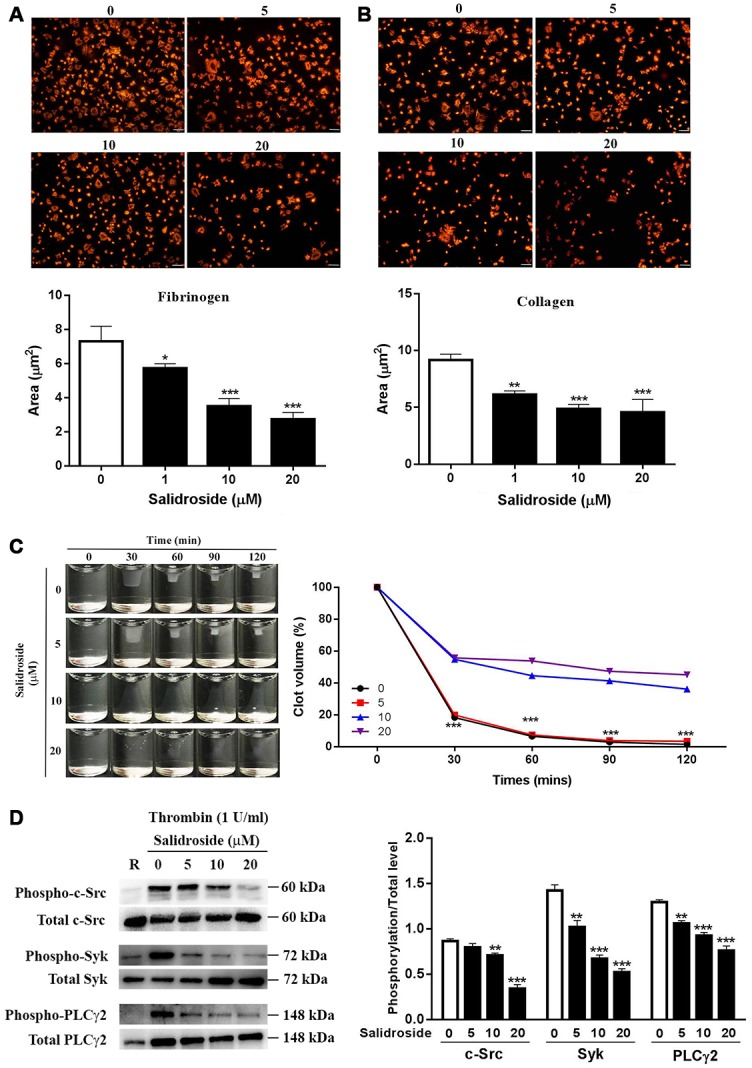
**Platelet spreading and clot retraction.** Washed human platelets were placed on glass coverslips coated with fibrinogen (**A**) or collagen (**B**) at 37°C for 90 min followed by staining with Alexa Fluor-546-labelled phalloidin (mean ± SD, n = 3). Clot retraction was also performed in Salidroside-treated platelets (mean, n = 3) (**C**). Meanwhile, under clot retraction condition, the phosphorylation level of c-Src, Syk and PLCγ2 was measured by western blot and represented as a ratio relative to the total level (mean ± SD, n = 3) (**D**). For panel A, B and D, data were analyzed by one-way ANOVA. Compared with 0, ^*^P < 0.05; ^**^P < 0.01; ^***^P < 0.001. For panel C, data were analyzed by two-way ANOVA. Compared with 10 or 20, ^***^P < 0.001.

Platelet spreading and clot retraction are regulated by early- and late-αIIbβ3 outside-in signaling respectively [5]. To assess whether salidroside plays a role in αIIbβ3 outside-in signaling transduction, we first measured platelet spreading and observed significantly inhibited platelet spreading on both fibrinogen (Figure 3A) and collagen (Figure 3B) after salidroside treatment in a dose-dependent manner compared with vehicle treatment. In addition, we also measured thrombin-mediated clot retraction. Consistent with platelet spreading, clot retraction was also significantly decreased in salidroside-treatment platelets compared with vehicle-treated platelets as demonstrated by significantly increased clot volume in salidroside-treated platelet than that in vehicle-treated platelets (Figure 3C). Since activation of αIIbβ3 outside-in signaling leads to phosphorylation of c-Src, Syk, and PLCγ2, and subsequent platelet spreading and clot retraction [25, 26], we then detected the phosphorylation status of these signaling proteins. In accordance with the decreased platelet spreading and clot retraction, salidroside-treated platelets exhibited significantly reduced phosphorylation of c-Src, Syk, and PLCγ2 (Figure 3D) after thrombin stimulation compared with vehicle-treated platelets.

### Salidroside-treated mouse platelets shows reduced platelet function

**Figure 4:**
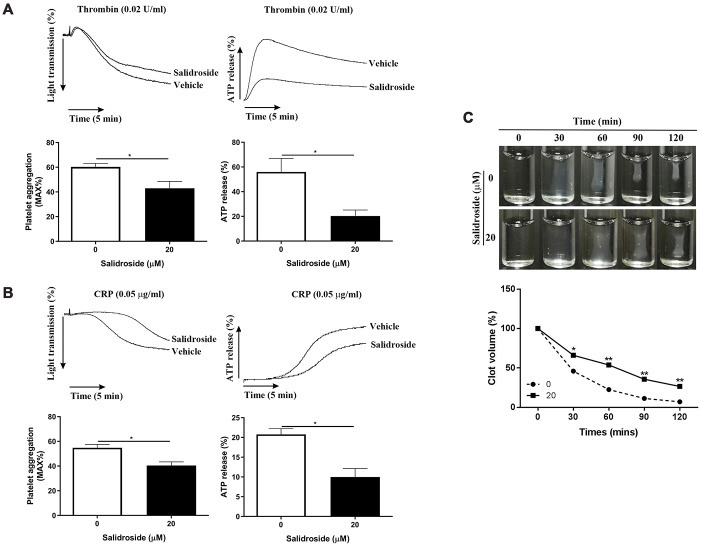
**Effect of salidroside on mouse platelet function.** Washed mouse platelets were treated with vehicle or 20 μM salidroside followed by measuring platelet aggregation and ATP release in response to thrombin (0.02 U/ml) (**A**) or CRP (0.05 μg/ml) (**B**) as well as thrombin-mediated clot retraction (**C**). For panel A and B, data were presented as mean ± SE (n = 3-4) and analyzed by unpaired student t-test. ^*^P < 0.05. For panel C, data were shown as mean (n = 3-4) and analyzed by two-way ANOVA. Compared with 0, ^*^P < 0.05; ^**^P < 0.01.

To evaluate whether salidroside also exerts the similar effects on mouse platelets, we isolated mouse platelets and treated them with salidroside. As seen in Figure 4, salidroside treatment significantly inhibited platelet aggregation in response to thrombin (0.02 U/ml) (Figure 4A) or CRP (0.05 μg/ml) (Figure 4B) compared with vehicle treatment. In addition, a significantly reduced ATP release was found in salidroside-treatment platelets after stimulation by thrombin or CRP. Furthermore, we also assessed the effect of salidroside on thrombin-mediated clot retraction and found that clot retraction was also significantly decreased after salidroside treatment compared with platelets treated with vehicle (Figure 4C). These data show that salidroside also inhibits platelet aggregation, ATP release and clot retraction in mouse platelets.

### Impaired hemostasis and arterial thrombosis *in vivo* after salidroside treatment

**Figure 5:**
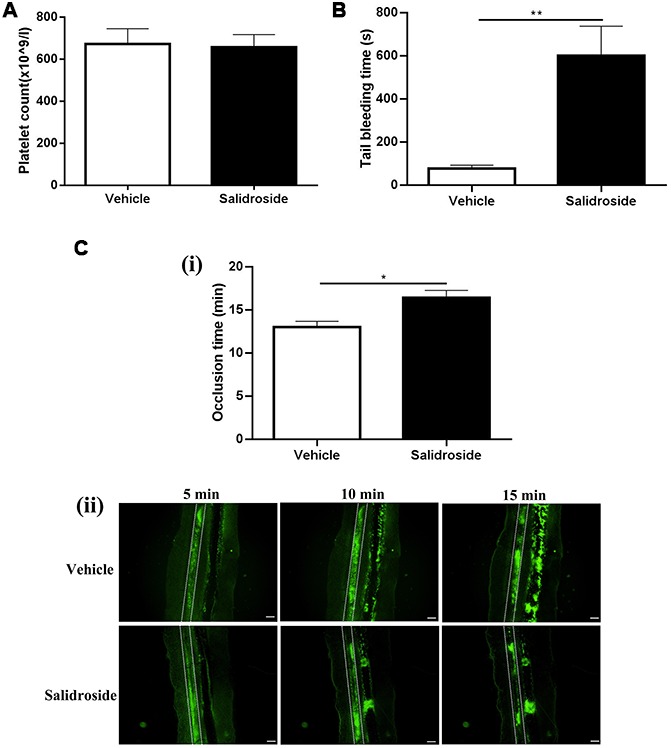
**Salidroside’s effect on hemostasis and arterial thrombosis in mice.** Mice were injected with salidroside (20mg/kg) intraperitoneally followed by analysis of platelet count (**A**) (mean ± SE, n = 7) and tail bleeding time (mean ± SE, n = 6) (**B**). Salidroside (20 μM) or vehicle-treated platelets were labelled with calcein and infused into salidroside-treated or vehicle-treated mice respectively followed by challenging with 10% FeCl_3_ to induce arterial thrombus formation. The vessel occlusion time was recorded (mean ± SE, n = 6) (**C**). Data were analyzed by unpaired student t-test. ^*^P < 0.05; ^**^P < 0.01.

As salidroside inhibits both human and mouse platelet function, we next investigated whether it affects the platelet function *in vivo* through intraperitoneal injection of salidroside (20 mg/kg) into mouse followed by analysis of tail bleeding time which could reflect the *in vivo* hemostasis. Before tail bleeding time was assessed, we first measured the platelet count after administration of salidroside to see if salidroside affects platelet production or turn over *in vivo* and found that there was no significant change of platelet count in mice receiving injection of either vehicle or salidroside (Figure 5A), indicating that salidroside does not affect platelet production or turn over. However, salidroside injection significantly prolonged the tail bleeding time compared with vehicle administration (Figure 5B). In addition, the arterial thrombus formation induced by FeCl_3_ was significantly inhibited in mice treated with salidroside compared with that in mice treated with vehicle as demonstrated by prolonged arterial vessel occlusion time in mice receiving salidroside injection (Figure 5C). These data indicate that salidroside impairs *in vivo* hemostasis and arterial thrombus formation.

### Decreased venous thrombus formation in mice treated with salidroside

**Figure 6:**
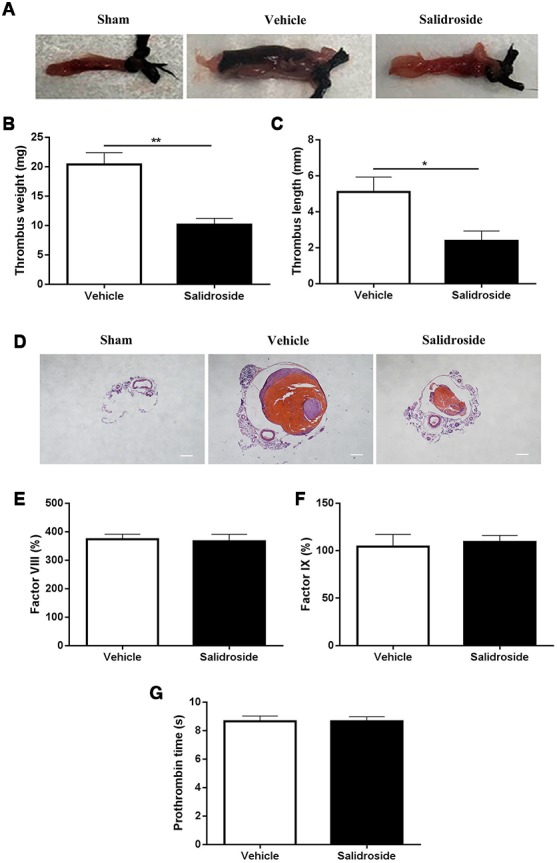
**Deep vein thrombus formation and coagulation analysis.** After intraperitoneal injection of salidroside (20 mg/kg) or vehicle, mice underwent ligation of inferior vena cava (IVC) to initiate venous thrombus formation. After 24 h, the IVC samples (**A**, representative IVC from 5 mice) were collected for measuring the thrombus weight (**B**) and length (**C**) (n = 5). Meanwhile, the histological assessment of the ligated IVC samples was also performed (magnification x 40, scale bar =1000 μm) (**D**). In addition, peripheral blood was collected from salidroside or vehicle treated mice for analysis of coagulation factor FVIII (**E**), FIX (**F**) and prothrombin time (**G**) (n = 7). Data were presented as mean ± SE and analyzed by unpaired student t-test.

Beyond hemostasis and arterial thrombosis, platelets also play a role in the venous thrombus formation [27]. Since salidroside inhibits *in vivo* platelet function, we next investigated whether it influences venous thrombus formation using deep vein thrombosis (DVT) model (stenosis of the inferior vena cava). As seen in Figure 6, venous thrombus formation was found in DVT mouse model (Vehicle) and not observed in sham group (Figure 6A). After administration of salidroside, the thrombus weight was significantly decreased (Figure 6B) and thrombus length was significantly shortened (Figure 6C) in DVT mouse model compared with vehicle treatment. In addition, H&E staining of the venous cross-section showed dramatic thrombus formation in DVT mouse model treated with vehicle as demonstrated by the presence of a red thrombus (Figure 6D), which was reduced after salidroside injection. To exclude the involvement of coagulation in deep vein thrombosis, we also measured the level of coagulation factor VIII (Figure 6E) and IX (Figure 6F) as well as the prothrombin time (Figure 6G) and found that there was no significant difference of these parameters in mice receiving the injection of vehicle and salidroside. Taken together, these data show that salidroside decreased deep vein thrombus formation without affecting coagulation.

### Salidroside reduces phosphorylation of AKT and GSK3β in platelets

**Figure 7:**
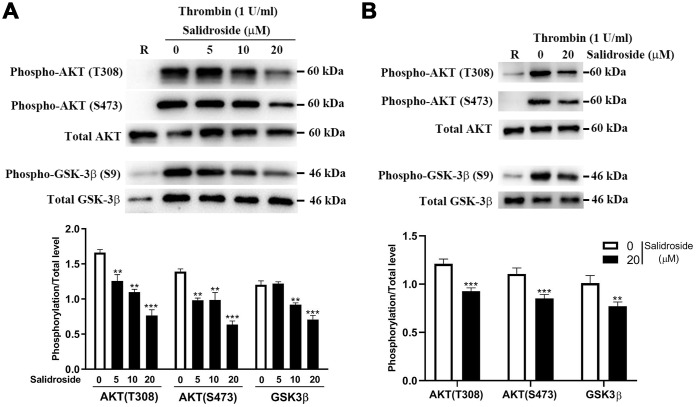
**Phosphorylation level of AKT and GSK3β.** After salidroside treatment, human (**A**) or mouse (**B**) platelets were treated with 1 U/ml thrombin for 15 min followed by analysis of the phosphorylation level of AKT and GSK3β by western blot. The protein expression was quantified using Image J software and represented as a ratio of phosphorylation to the total level (mean ± SD, n = 3). Data were analyzed by one-way ANOVA. Compared with 0, ^**^P < 0.01; ^***^P < 0.001.

As salidroside has been shown to affect cancer cell biological behaviors through regulating AKT signaling [28, 29], we measured whether salidroside could affect AKT signaling in platelets through measuring the phosphorylation of AKT and found that salidroside treatment significantly decreased the phosphorylation of AKT (T307/S483) in both human (Figure 7A) and mouse (Figure 7B) platelets after stimulation by thrombin, indicating that salidroside also plays a role in AKT signaling in platelets. As glycogen synthase kinase 3 (GSK3) is the first identified substrate of AKT, we next detected the phosphorylation of GSK3β (the highly expressed isoform in platelets) in salidroside-treated platelets after stimulation. As seen in Figure 7, a significantly decreased phosphorylation of GSK3β (Ser9) was observed in platelets after salidroside treatment in both human and mouse platelets. Taken together, these data indicate that salidroside inhibits platelets function possibly through downregulation of AKT signaling.

### Inhibition of GSK3β reversed the inhibitor effect of salidroside on platelet function

**Figure 8:**
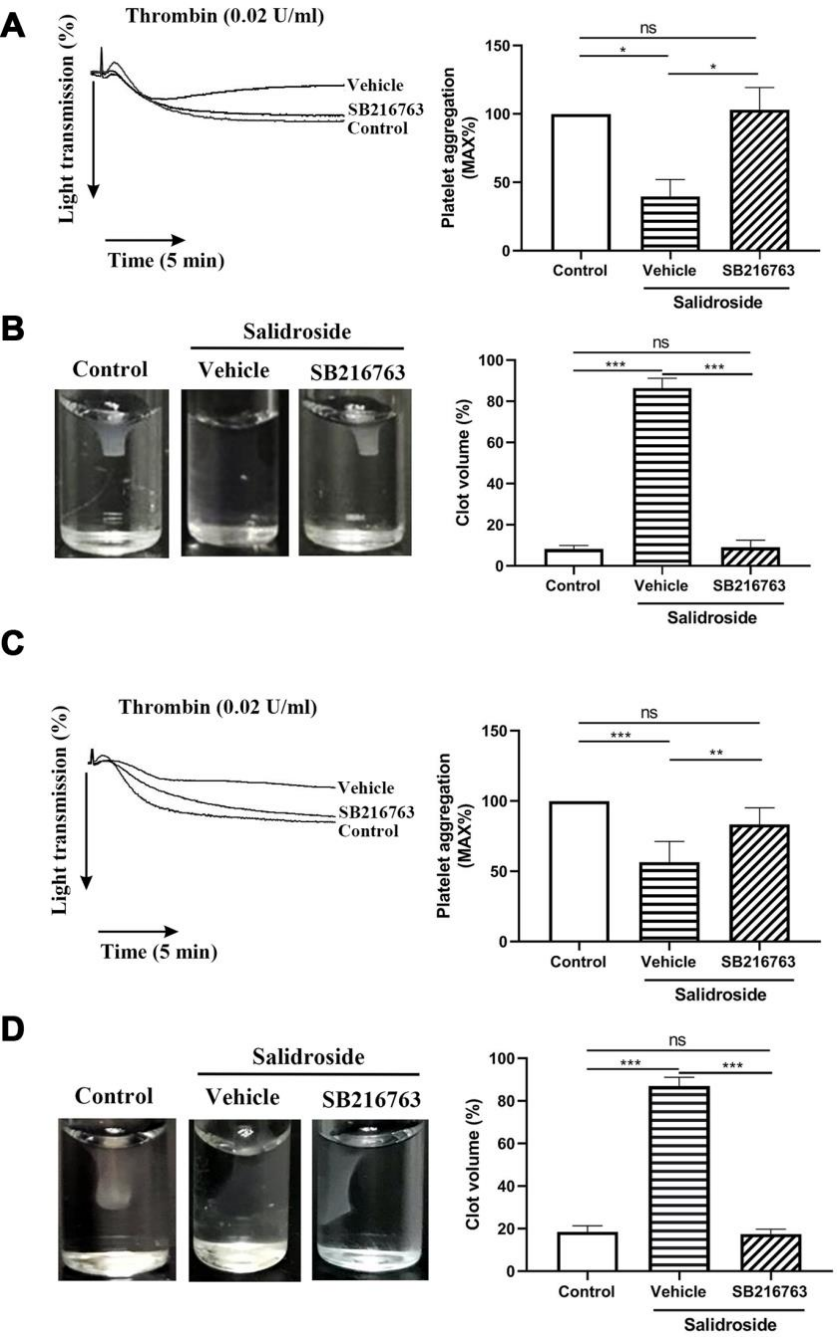
**Effect of inhibition of GSK3β on platelet function.** Washed human (**A** and **B**) or mouse (**C** and **D**) platelets were pretreated with GSK3β inhibitor SB216763 (10 μM) for 2 h at 37C followed by treated with salidroside (20 μM) at 37 for 1 h. After that, platelet aggregation in response to thrombin (**A** and **C**) and clot retraction (**B** and **D**) was performed. The clot image was captured at 60 min after initiation. Data were shown as mean ± SE (n = 4) and analyzed by one-way ANOVA. *P < 0.05; **P < 0.01; ***P < 0.001. ns: not significant.

GSK3β kinase activity is inactivated after phosphorylation of Ser9 [30, 31], and decreased GSK3β phosphorylation in salidroside-treated platelets might cause increased GSK3b kinase activity, which negatively regulates platelet function [32]. To further investigate whether salidroside impairs platelet function through AKT/GSK3β signaling, we used GSK3β inhibitor (SB216763) to pretreat platelets prior to salidroside treatment and found that inhibition of GSK3β full reserved the inhibitory effect of salidroside on platelet aggregation and clot retraction in both human (Figure 8A, 8B) and mouse (Figure 8C, 8D) platelets, confirming that salidroside inhibits platelet function through regulation of AKT/GSK3β signaling.

## DISCUSSION

Salidroside has been demonstrated to possess several biological and pharmacological properties [13, 14] and plays a protective role in several disease models such as diabetic Goto-Kakizaki Rats [15] and atherosclerotic plaque formation in mice [18]. Since salidroside exerts broad spectrum activities under pathological conditions, the effect of salidroside on platelet function is unclear. In our study, we assessed the role of salidroside in platelet function and demonstrated that salidroside impairs platelets function and inhibits both arterial and venous thrombus formation as well as the hemostatic function of platelets. As a main modulator of thrombosis and hemostasis, platelet has been shown to contribute to the early development [33] and the late events of atherosclerosis, such as rupture of the vulnerable plaque and arterial thrombus formation [34, 35]. Considering the inhibitory effect of salidroside on platelet function, we speculate that salidroside might be used to treat atherosclerosis through affecting platelet function. However, we did not perform the atherosclerosis mouse model to test this hypothesis, which would be a limitation of our study.

Platelet activation is initiated mainly through binding of the surface glycoprotein receptors, such as α_IIb_β_3_, GPIbα and GPVI to their respective ligands [36] and these platelet receptors are critical for maintaining of the normal functions of platelets [37]. Abnormality of these platelet receptors either with reduced expression or impaired function can cause severe bleeding disorders [38]. At sites of vascular injury, platelet GPIbα and GPVI adhere to the damaged blood vessel wall through binding to VWF and collagen respectively, causing intracellular signaling pathway transduction and α_IIb_β_3_ activation, which regulates platelet aggregation and thrombus formation [1, 4]. As salidroside inhibits platelet aggregation and thrombus formation, we tested whether salidroside affects platelet receptors expression and observed that there were no changes of platelet receptors expression in salidroside- and vehicle-treated platelets, indicating that salidroside impairs platelet function without affecting platelet glycoprotein receptors expression.

AKT is a serine/threonine protein kinase and can be activated by several growth factors or cytokines which is predominantly dependent on phosphoinositide 3-kinase (PI3K), which phosphorylates phosphoinositides (PIs) to produce PI(3,4,5)P3 (PIP3) [39]. After binding to PIP3 through an N-terminal PH (pleckstrin homology) domain, AKT undergoes translocation to the plasma membrane, leading to phosphorylation at residue Thr308 and Ser473 [40, 41]. Both of these sites phosphorylation is necessary for the full induction of AKT kinase activity. Once activated, AKT can phosphorylate multiple substrates, including protein kinases, transcription factors, and cell cycle regulators, thus playing an important regulatory role in cell proliferation, cell cycle, survival, apoptosis [39]. As anucleated cells, platelets also express AKT which involves in regulating platelet function, such as platelet aggregation, activation, granule secretion as well as integrin signaling [42, 43]. Through inhibition of PI3K/AKT signaling pathway, salidroside have been shown to repress proliferation, migration and invasion of human lung cancer cells [29], induce autophagy in human colorectal cancer cells [28] suppress LPS-induced myocardial injury [44] as well as attenuate Adriamycin-induced focal segmental glomerulosclerosis [45]. Consistent with these, in our present study, we demonstrated that salidroside treatment significantly reduced the phosphorylation of AKT (Thr308/Ser473) after stimulation with thrombin in both human and mouse platelets, indicating that salidroside exerts antiplatelet activities possibly through inhibition of AKT signaling. As several studies have demonstrated that some specific microRNAs can interact with AKT and its downstream signaling pathway, such as miR-16 [46], miR-126 [47] and these microRNAs are expressed in platelets and play a regulatory role in platelet activation [48, 49], whether salidroside impairs AKT signaling through regulating the expression of microRNAs in platelets remains unclear and requires further investigations in the future.

The Ser and Thr protein kinase glycogen synthase kinase 3 (GSK3) was the first AKT substrate identified and consists of two isoforms, GSK3α and GSK3β [50]. GSK3 is active in resting conditions and becomes inactivated upon stimulation. AKT exerts an inhibitory role in GSK3 kinase activity through phosphorylation of residue on Ser21 (GSK3α) or Ser9 (GSK3β) [30, 31], leading to decreased GSK3 activity and the release of a tonic inhibition of the GSK3 substrate [51–53]. Both GSK3α and GSK3β are present in platelet and are phosphorylated upon stimulation with GSK3b being highly expressed GSK3 isoform [32, 54, 55]. Using GSK3β^+/-^ mice, a previous study demonstrated that GSK3β negatively regulates platelet function and thrombus formation [32]. In this study, we found that salidroside treatment significantly decreased GSK3β phosphorylation (Ser9) in both human and mouse platelets after stimulation with thrombin, indicating that salidroside could not inhibit GSK3 kinase activity due to reduced phosphorylation of AKT, resulting in decreased platelet function. To further evaluate the role of GSK3β in platelet function after salidroside treatment, we pretreated platelets with GSK3β inhibitor followed by incubation with salidroside and found that addition of GSK3β inhibitor could reverse the inhibitory effect of salidroside on platelet aggregation and clot retraction in both human and mouse platelets, suggesting that salidroside inhibits platelet function through AKT/GSK3β signaling.

In conclusion, salidroside impairs platelet function, *in vivo* hemostasis, arterial and venous thrombus formation through inhibition of AKT/GSK3β signaling, suggesting that it may be a novel therapeutic drug for treating thrombotic or cardiovascular diseases.

## MATERIALS AND METHODS

### Reagents

Salidroside was purchased from MedChemExpress (Monmouth Junction, NJ, USA) with a purity ≥ 98%. Collagen-related peptide (CRP) was prepared as previously described [56]. Thrombin (≥ 10 NIH units/vial) were from Chrono-log Corporation (Havertown, PA, USA). FITC-conjugated mouse anti-human CD41a and PAC-1 antibody were from BD Biosciences (San Jose, CA, USA) and BECTON DICKINSON (San Jose, CA, USA) respectively. PE-conjugated anti-human/mouse CD62p (P-Selectin) and anti-human Glycoprotein VI purified antibody were purchased from eBioscience (San Diego, CA, USA). FITC-conjugated anti-CD42b antibody was from Abcam (Cambridge, MA, USA). FITC-conjugated goat anti-mouse IgG was purchased from ZSGB-BIO (Beijing, China). β-actin antibody and anti-rabbit IgG (HRP-linked) antibody were purchased from Cell Signaling Technology (Danvers, MA, USA). SB216763 (GSK-3β inhibitor) was purchased from ApexBio Technology.

### Animals

C57BL/6 mice with an age of 8-10 weeks and weight of 24-28g were bought from SLAC Laboratory Animal Co., Ltd. (Shanghai, China). Mice were housed in specific pathogen free (SPF) grade environment ensuring 12 h light/dark cycle with free access to food and water. Experiments on mice were performed in accordance with ARRIVE guidelines and the National Institutes of Health guide for the care and use of Laboratory animals (NIH Publications No. 8023, revised 1978). The study was approved by the ethnic committee of Xuzhou Medical University.

### Platelet preparation

All experimental procedures involving collection of human and mouse blood were approved by the Ethic Committee of Xuzhou Medical University. Informed consent was acquired from all participants prior to the study. Platelets were isolated from human and mouse blood as described previously [57, 58]. For human platelets, ACD-anti-coagulated venous blood was centrifuged for 20 min at 120 х *g* at room temperature to obtain platelet-rich plasma (PRP) which was centrifuged at 1,350 х *g* for 15 min, washed three times with CGS buffer and resuspended in Tyrode’s buffer. Mouse platelets were isolated from ACD anti-coagulated blood and resuspended in Tyrode’s buffer.

### Salidroside treatment

Isolated human or mouse platelets were treated with different doses of salidroside (0, 5, 10 and 20 μM for human platelets; 0 and 20 μM for mouse platelets) at 37°C for 1 h. For some experiments, isolated human or mouse platelet were incubated with SB216763 (10 μM) or vehicle for 2 h at 37 °C and then treated with salidroside followed by analysis of relevant analysis.

### Platelet aggregation and ATP release

After salidroside treatment, human platelet aggregation in response to thrombin (0.03 U/ml) or CRP (0.1 μg/ml) was assessed in a Lumi-Aggregometer Model 700 (Chrono-log Corporation, Havertown, PA, USA) at 37°C with stirring (1000 rpm). ATP release was monitored in parallel with platelet aggregation after addition of luciferin/luciferase reagent (Chrono-log Corporation) in accordance with the manufacturer's instructions. ATP release was presented as a percentage relative to the vehicle (0 μM salidroside) treatment. For mouse platelet aggregation, thrombin (0.02 U/ml) or CRP (0.05 μg/ml) was used.

### Platelet α-granule release

Platelet α-granule release (P-selectin surface expression) in platelets stimulated with thrombin (0.03 and 0.1 U/ml) or CRP (1 and 2 μg/ml) was measured using PE-conjugated anti-P-selectin antibody by flow cytometry [59].

### Surface expression of platelet receptors

The platelet receptors expression was measured using FITC-conjugated anti-CD42b antibody (GPIbα), FITC-conjugated mouse anti-human CD41a antibody (α_IIb_) and anti-human GPVI antibody (detected by FITC-conjugated goat anti-mouse IgG) by flow cytometry.

### Platelet spreading

Glass coverslips were pre-coated with fibrinogen (10 μg/ml) or collagen (10 μg/ml) at 4°C overnight and human platelets were placed on them at 37°C for 90 min followed by fixation, permeabilization and staining with Alexa Fluor-546-labelled phalloidin. Platelet spreading was detected under a fluorescence microscopy (Nikon-80i) using an X100 oil objective. The surface coverage was quantified using Image J software.

### Clot retraction

Clot retraction was performed by addition of thrombin (1 U/ml), 2 mM Ca^2+^ and 0.5 mg/ml fibrinogen at 37°C as described previously [57, 58]. Images were captured every 30 min.

### Tail bleeding assay

Salidroside (20 mg/kg) was intraperitoneally injected into mice followed by analysis of tail bleeding time after 30 min as described previously [57, 58].

### FeCl_3_-induced arterial thrombosis

Salidroside (20 μM) or vehicle-treated mouse platelets (1 x 10^8^) were labelled with calcein and administrated into salidroside-treated mice or vehicle-treated mice respectively via tail vein injection. After 30 min, mesenteric arterioles were challenged with 10% w/v (final concentration) FeCl_3_ to induce thrombus formation which was monitored by a fluorescence microscopy (Olympus BX53) [57, 58].

### Deep vein thrombosis

After anesthesia with isoflurane-oxygen mixture, an incision was performed in the midline of the abdomen followed by exteriorization of intestines which were then soaked in warm saline throughout the whole process to prevent drying out. The inferior vena cava (IVC) was ligated with a 2-0 nonabsorbable suture. At 24 h following IVC ligation, thrombosis was assessed and thrombi were excised for measurement of weight and length.

### H&E staining

Thrombi were excised from mice with DVT at 24 h after IVC ligation and fixed with 4% paraformaldehyde, dehydrated and paraffin embedded. The slicer was sectioned and sliced to a thickness of 10 μm, followed by H&E staining and analysis of results under a light microscopy.

### Coagulation analysis

Plasma was extracted from salidroside or vehicle-treated mice and the level of factor VIII and X, and prothrombin time was detected on an automated coagulation analyzer (Sysmex CS-5100).

### Western blotting

Levels of total and phosphorylated c-Src (anti-Tyr-416, Cell Signaling Technology; pan-c Src, Proteintech), Syk (anti-Tyr-525 and pan-Syk, Bioworld Technology), PLCγ2 (anti-Tyr-1217 and pan-PLCγ2, Bioworld Technology), AKT (anti-Thr308 and anti-Ser473, Cell Signaling Technology; pan-AKT, Affinity Biosciences), or GSK-3β (anti-Ser9, Cell Signaling Technology; pan-GSK-3β, Affinity Biosciences) were assessed by SDS-PAGE/western blot. The density of protein band was quantified using Image J software and the phosphorylation level was shown as a ratio to the total level.

### Statistical analysis

Data are analyzed by GraphPad Prism software and represented as mean ± standard deviation (SD) or mean ± standard error (SE). Student t-test was performed for comparison of two groups. Comparison of difference among multiple groups was assessed by one-way ANOVA and comparison among different groups over time was conducted by two-way ANOVA with Bonferroni post-hoc analysis. P < 0.05 indicates statistically significance.
